# Care for ill-housed persons: access and professional proactivity in primary care

**DOI:** 10.1590/0034-7167-2023-0385

**Published:** 2025-08-25

**Authors:** Lucas Alves Gontijo, Bruna Moreira da Silva, Marcelo Pedra Martins Machado, Daniela Priscila Oliveira do Vale Tafner, Edilene Aparecida Araújo da Silveira, Valéria Conceição de Oliveira, Eliete Albano de Azevedo Guimarães, Selma Maria da Fonseca Viegas

**Affiliations:** I Universidade Federal de São João del-Rei. Divinópolis, Minas Gerais, Brazil.; II Fiocruz Brasília. Brasília, Distrito Federal, Brazil.; III Universidade Regional de Blumenau. Blumenau, Santa Catarina, Brazil.

**Keywords:** Ill-Housed Persons, Primary Health Care, Health Services Accessibility, Health Personnel, Equity in Access to Health Services, Personas con Mala Vivienda, Atención Primaria de Salud, Accesibilidad a los Servicios de Salud, Personal de Salud, Equidad en el Acceso a los Servicios de Salud

## Abstract

**Objectives:**

to understand access to primary care for ill-housed persons and professional proactivity in municipalities without a Street Outreach Office.

**Method:**

a qualitative approach study, under the Grounded Theory methodological framework and Symbolic Interactionism theoretical framework, carried out with 30 Family Health Strategy professionals and six key informants from the Socio-Assistance Network, using open-ended interviews and memos.

**Results:**

the lack of planning for health care for ill-housed persons was evident. Access occurs through spontaneous demand and, almost always, under the intervention of Social Assistance Network professionals. Intersectoral and proactive professional work were highlighted as axes in overcoming inequities and guaranteeing access.

**Final considerations:**

the need to expand access is ratified, with identification, registration and monitoring of ill-housed persons in primary care. It is necessary to encourage professional proactivity, given their notoriety in strengthening equitable actions.

## RESUMEN

**Objetivo:**

comprender el acceso a la atención primaria de personas con mala vivienda y la proactividad de los profesionales en municipios sin Clínica de Calle.

**Método:**

estudio cualitativo, utilizando el marco metodológico de la Teoría Fundamentada y el marco teórico del Interaccionismo Simbólico, realizado con 30 profesionales de la Estrategia Salud de la Familia y seis informantes clave de la Red de Asistencia Social. Se utilizaron entrevistas abiertas y memorandos.

**Resultados:**

se evidenció la falta de planificación para la atención de la salud de las personas con mala vivienda. El acceso se produce mediante la aceptación de una demanda espontánea y, casi siempre, mediante la intervención de profesionales de la Red de Asistencia Social. Se destacaron la intersectorialidad y la proactividad profesional como ejes para superar las inequidades y garantizar el acceso.

**Consideraciones finales:**

se confirma la necesidad de ampliar el acceso, con identificación, registro y seguimiento de las personas con mala vivienda en la atención primaria. Es necesario fomentar la proactividad de los profesionales, dada su notoriedad en el fortalecimiento de acciones equitativas.

## INTRODUCTION

As a preferential gateway to the Brazilian Health System (In Portuguese, *Sistema Único de Saúde* - SUS), Primary Health Care (PHC) organizes the flow of persons in the Health Care Network (In Portuguese, *Rede de Atenção à Saúde* - RAS), in order to ensure that care is offered close to each person’s home. PHC has the ability to meet local specificities and regional characteristics, such as the presence of itinerant and dispersed populations in the coverage area, thus ensuring the effectiveness of health equity^(1)^.

Health is a fundamental right of citizenship, and this implies that the State ensures access to healthcare actions and services for everyone through social and economic policies. However, many excluded persons continue to fight for the right to actively participate in society, as is the case of homeless persons, who experience stigma and prejudice, on the margins of social processes. Moreover, they experience varying degrees of violations and vulnerabilities^(2,3)^, which are factors that contribute to the legitimization of violence against the identity of these persons^(4)^. In this regard, to ensure broad, simplified and safe access to public policies, the Brazilian National Policy for Home-less Persons (In Portuguese, *Política Nacional para Pessoas em Situação de Rua* - PNPSR)^(5)^ was established in Brazil in 2009, representing an advance in the care of these persons through intersectoral actions^(4)^, in addition to promoting the principles of equity, humanization and universality, recognizing the uniqueness and respect for human dignity and citizenship of these persons^(6)^. According to the nomenclature adopted in Brazil, ill-housed persons, in the Health Sciences Descriptors and Medical Subject Headings, will be referred to in this study as “persons living on the streets”.

**Figure 1:**
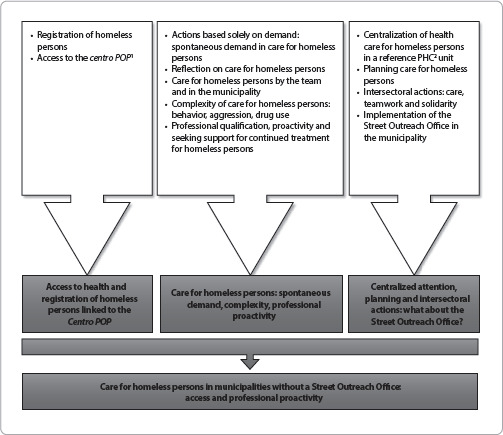
Category “Care for homeless persons in municipalities without a Street Outreach Office: access and professional proactivity”, subcategories and codes *in vivo*, two municipalities in the Midwest region of Minas Gerais, Brazil, 2022

*Note:^1^Specialized Reference Center for the Homeless Population;^2^Primary Health Care.*

In order to help overcome inequities and expand access to healthcare for homeless persons, in 2011, the Ministry of Health established the Street Outreach Office team (SOOt), which aims to expand responses to the demands and needs of these persons in an itinerant manner and integrated with the Family Health Strategy (FHS) teams and, when necessary, with other healthcare points, according to their unique demands^(7)^. However, despite the advances in the rights acquired by homeless persons, they continue to face obstacles to access healthcare, especially in municipalities without SOOt, a factor that generates exclusion, leaving access to emergency care units^(2)^. Thus, collaboration with intra and intersectoral networks is necessary in order to promote equity and improvements in living conditions and lifestyles on the streets, with a reduction in harm and health risks^(8)^.

In recent decades, homeless persons have become a global public health and political problem. A study conducted in Austria, Greece, Spain and the United Kingdom showed that the relationship between these persons and health is complex, because homelessness is the cause of health problems and their complications, which in turn increase the time spent on the streets. These persons die earlier than the general population and have less access to PHC, as they use emergency services more often and do not always receive adequate treatment and monitoring for chronic diseases. It is urgent to guarantee access, care and meet the needs of persons living on the streets, instead of facing a complex, segregating and exclusionary healthcare system^(9)^.

Given the complex reality of homeless persons, marked by exacerbated social and clinical needs, in Buffalo, New York, a collaborative, intersectoral, interagency and multidisciplinary team was created to support transition of care for these persons upon hospital discharge through medical care and a respite program. The study indicates that the traditional organizational model of healthcare services is insufficient and a new paradigm is needed that transcends curative practices and embraces an intersectoral and proactive approach, prioritizing collaboration between different sectors and the proactive action of the professionals involved^(10)^.

## OBJECTIVE

To understand access to primary care for ill-housed persons and professional proactivity in municipalities without a Street Outreach Office.

## METHODS

### Ethical aspects

The research complied with the ethical aspects of Resolutions 510/2016 and 580/2018 of the Brazilian National Health Council. In order to guarantee participant privacy, the Informed Consent Form was signed after pertinent information about the research and the ethical precepts^(11,12)^. The project was approved by the Research Ethics Committee, under Opinion 5.173.172. Participants were identified with an alphanumeric code (I1, I2, 13...)

### Study design and theoretical-methodological framework

This is a qualitative study, resulting from a master’s dissertation, which had Grounded Theory (GT)^(13)^ as its methodological framework and Symbolic Interactionism (SI)^(14)^ as its theoretical framework.

GT^(13)^ provides a combination of results that allows us to explain phenomena, as well as discern, understand and form an action plan or theory through the use of the data collected^(12)^. SI makes it possible to understand how individuals perceive others through their actions and interactions and how they collectively give new meaning to their experiences. It is based on three premises, which focus on understanding the social world from persons’ perspectives, namely: social action is mediated by meanings, and persons act based on the meaning that things have for them; meanings emerge from social interaction and meanings are formed during interaction; meanings are dynamic and interpretative, i.e., they are constantly changing, and can be reinterpreted according to the context and the situation^(14)^.

To meet the criteria for reporting qualitative research, the researchers adopted the COnsolidated criteria for REporting Qualitative research (COREQ).

### Methodological procedures

PHC health unit selection occurred through a random draw, except for those that refer to persons living on the streets, which were included without a draw. After the initial approach of participants in person at the workplace and obtaining Informed Consent Form, the interview was carried out. Thirty PHC professionals and six key informants, who are professionals from institutions that shelter persons living on the streets, with at least one year of experience in the position, participated in this study. Professionals who were away from work or on vacation were excluded. The interviews took place in person at the health units and reception institutions in a reserved room, after participants’ voluntary acceptance and subsequent scheduling for data collection, with the number of participants determined by theoretical data saturation, i.e., saturation of the concepts/meanings arising from the raw data^(13)^, totaling 36 research participants.

### Study setting

The study setting consists of two medium-sized municipalities, belonging to the Western Macroregion of the state of Minas Gerais (MG), Brazil, without SOOt. Population size was a decisive factor in the selection process. The two largest cities in the region were chosen, according to the numbers estimated by the Brazilian Institute of Geography and Statistics (In Portuguese, *Instituto Brasileiro de Geografia e Estatística* - IBGE) in 2022.

### Data source

Open-ended, intensive interviews were used, with a semi-structured script and recorded in memos.

### Data collection and organization

Data collection took place between February and November 2022. The interview was conducted by the main researcher together with a scientific initiation student, audio-recorded and transcribed in full with concomitant analysis, in accordance with the GT premise. The interview was validated by the research participant after reading transcription, and all participants in this study allowed the use of their interview data in its entirety. The interview was guided by a semi-structured script with nine questions that addressed healthcare for homeless persons, in order to encourage participants’ reflection on the experiences lived and related to the object under study. With a flexible approach, it was possible to deepen the concepts/meanings for developing the theory. The mean duration of interviews was 20 minutes, ranging from 13 to 42 minutes, according to the freedom of expression when addressing the topic and in view of the unique experiences lived.

### Data analysis

Data analysis was carried out in four stages, such as open, axial, selective and process coding, allowing greater flexibility in interpretation and in deepening meanings, based on the realities experienced^(13)^.

In open coding, in a dynamic and fluid process, interviewees’ statements made it possible to reveal, name and develop concepts, which originated the *in vivo* codes and properties. In axial coding, the data obtained in open coding were subcategorized and incorporated into subthemes^(13)^, determining five categories: 1) Spontaneous demand, complexity of care and professional proactivity in municipalities without a Street Outreach Office; 2) Exclusion, prejudice and invisibility of homeless persons, refuting the right to health; 3) Equity in tackling inequities: welcoming homeless persons; 4) Difficulties in implementing self-care strategies for homeless persons; 5) In Primary Care, the encounter with homeless persons: what are they like and why did they leave home? In selective coding, the theory was integrated and refined in order to represent the research results, thus assuming the central category “Care for homeless persons in municipalities without a Street Outreach Office: from access to tackling inequities”, also called theory. During all these stages, coding for the process occurred, which consisted of going back and forth through the data until the moment of constructing the central category, and the data were challenged and analyzed repeatedly throughout the analysis process^(13)^.

## RESULTS

Thirty-six professionals were interviewed, including seven nurses, one nursing assistant, three nursing technicians, three dentists, one oral health technician, two oral health assistants, four doctors, nine Community Health Workers (CHWs), three psychologists, one oral health attendant, one social caregiver and three social workers. Of these, 25 were female and 11 were male, with a mean age of 39 years, with a standard deviation of 22 and 59 years, and the time of experience in the area varied from 1 to 28 years.

This article addresses the category “Care for homeless persons in municipalities without a Street Outreach Office: access and professional proactivity”. As seen in Figure 1, it is possible to identify the *in vivo* codes/properties, subcategories and category that gave rise to this article.

Care for homeless persons presents singularities in the daily routine of PHC in both scenarios of this study.

“Open door”, “it is free demand”, “if they arrive, we welcome them” are expressions used by the research participants, emphasizing that health actions aimed at persons living on the streets are geared towards spontaneous demand in response to a specific need:

*In fact, there is no specific service, because it is an open door* [...] *but there is no service to go on visits! Because they have no place to live. So, when they feel a need for health, for care, it is an open door, and they come to the unit. You can’t deny it.* (I15)

*It’s the open door. It’s their free demand. When they need something from us, we provide it to them. They come, they ask, and we provide the help they need.* (I16)

Access is provided by responding to spontaneous demand, at pre-established times, sometimes in acute circumstances:

*The demand here is actually spontaneous* [...] *because they only seek help when they are in a very precarious situation, when they are in pain, like in the case of toothache, or when they have a fall, or when they get into trouble that hurts, when they have to take care of a wound, a fracture, something like that. They rarely come of their own free will.* (I26)

PHC welcoming is made possible through referrals made by Social Assistance Network professionals or in prior communication with the service to which users are referred.

*Homeless persons are usually seen with a referral.* [...] *they are always scheduled for that day. So, the service is easy, they won’t have to wait long. If there is no space available that day, we try to schedule the closest one.* (I20)

Thus, it is noted that healthcare is provided in a centralized manner in certain PHC teams in the municipalities, given the existence of social assistance equipment in the coverage area that connects homeless persons to PHC services:

*Here, the flow of care belongs to an area, so the micro-area where the Centro POP is located has a CHW who has direct contact there, has the medical records and, in general, they are referred by the social worker of the Centro POP, or they call here at the unit and bring the demand.* (I8)

*Basically, here, there are two ways, spontaneous demand, which is the open door, and there is the Shelter House, which is here in our coverage area* [...] *when she* [social worker] [...] *sees that she needs some healthcare, she gets in touch with us, and we normally schedule it or she refers it with a simple referral.* (I31)

According to participants in this study, in their daily lives, they face significant obstacles when caring for homeless persons, due to the lack of a history of longitudinal care and the fact that interventions often occur in tense situations due to the defensive behavior of these persons and the effects of psychoactive substances. These obstacles make it difficult to articulate and construct daily care plans for these persons in the context of PHC.

*So, the biggest difficulty I see is the first contact with them, the aggressiveness they show. They think we are prejudiced against them.* (I3)

*It’s a difficult group of persons. Sometimes, they already arrive here with some changes; most of them will have a psychiatric illness; 99% will have some type of addiction: alcohol, drugs... so it’s naturally difficult.* [...] *many persons already arrive altered, so how are you going to approach this group? It’s difficult, the person already arrives altered, you don’t have a history, a person arrives with a report that their medication was stolen, or disappeared, so how do you follow up on that?* [...] *there are multiple factors. It’s difficult to do longitudinal monitoring.* (I31)

Reflections are expressed on improving care for homeless persons, and strategies are outlined to expand access and improve healthcare for these persons, through proactive professional attitudes that minimize health inequities.

*Make a group effort to go to this little square, with a doctor and a nurse.* [...] *if a team were formed to go to meet them, one day, once a month, to where they are located, it would help a lot.* (I15)

*Health goes to where users are, because, often, we are unable to make them aware of going to FHS. Perhaps this would be the way to achieve equity.* (I18)

*In terms of health, we could improve projects. I don’t know, put an ambulance at night, where they gather more... measure blood pressure, glucose. See how they are...* (I34)

Despite the complexity of providing care to homeless persons, it is clear from participants’ statements that professional proactivity provides intra- and intersectoral links in overcoming barriers and ensuring access to care. This fact is evident in the routine of services, when these persons are included in the daily routine of PHC, however, with regard to registering persons in PHC, the discourses differ between participants and the municipalities.

*He has his medical records in the unit,* [...] *but he is not registered. He is not linked to a family.* (I7)

*It’s like a POP family, you know?* [...] *they are registered there as a family.* (I13)

*According to our working method, we register families according to their address, and these persons have no way of doing this.* (I16)

The goal is to ensure that each person has a dignified life, including health, food and shelter. In this regard, the Specialized Reference Center for the Homeless Population (*Centro POP*) was mentioned several times by survey participants:

*So, today you can stay at home* [Centro POP] *or you can go there to take a shower, eat and go back to the street.* (I1)

*There is the POP, which welcomes these homeless persons, follows them up in specialized consultations, I mean, consultations at the polyclinic.* (I7)

Each municipality without a SOOt has a routine and practice for assisting homeless persons, which infers the guarantee of access to health through social assistance programs that establish intersectoral relationships with health units closest to the shelter institution, but it provides for the centralization of care in a single unit, or in a more central unit in the municipality, where homeless people circulate as a“point of reference” in providing health care to these people (memorandum).

*They centralized the care for the homeless population in the unit that is within the scope of the Centro POP. So, today, the care for the homeless population that is registered with the Centro POP is provided by our health unit.* (I8)

*Here, we have a Shelter House* [Social Assistance Reference Center] *in the center, so when this user arrives there and needs assistance, then the house itself refers them here.* (I28)

In relation to the planning of healthcare for homeless persons, it was common in both realities to carry out specific actions according to the needs demanded by them and by social assistance services, operating responses to specific complaints, abstaining from the development of planned actions in order to promote these persons’ health, which may impact on the factors that interfere with quality of life and health.

*There is no planning. It doesn’t exist. It depends on their needs when they seek the unit. But does the unit do anything to promote it? It doesn’t.* (I15)

*There is no way to plan actions with this population. It is a very fluctuating, very irregular population.* [...] *we have already done it, going to the Shelter House,* [...] *giving them a talk about self-care, hygiene, oral hygiene. That is what we have managed to do so far,* [...] *there are no specific programs to go frequently, no. We went because they invited us.* (I33)

However, even without planning for healthcare for home-less persons, it is clear that intersectoral actions are extremely important in the construction of the care offered, given that PHC teams are engaged and supportive in serving these persons.

*Specifically, here at the Centro POP, which is a social assistance service, regarding health issues, we have more of an idea of support, we make referrals to PHC to the hospital...* [...] *we build a project, a monitoring plan together with users. If we identify a health issue, it is that issue of support. We support the person, often, we accompany persons to the exams they need to do, to the care they need to receive at PHC, the referrals to other services, such as CAPS.* (I17)

*Here, we have a lot of ease in terms of health. We have a very good partnership with the persons at the health center there, everything we need, they are attended to, they are referred there.* [...] *there is also CAPS-AD monitoring, some here attend, not all, and when they do attend, they have support from CAPS-AD, but it is very calm. When it is an emergency case, we call SAMU, they come.* (I36)

One of the municipalities is in the process of SOOt accreditation. Given this reality, professionals were concerned and hopeful about the implementation of the team, due to the possibility of expanding access and the potential for longitudinal monitoring:

*The Street Outreach Office has already been announced in the press* [...] *it would be an absolutely important unit for monitoring them.* [...] *I fear that the Street Outreach Office will become a large outpatient clinic, without psychosocial links, without links to the territory, without links to income generation, without other possibilities that this population would have in the territory.* (I22)

*We have been conducting surveys and discussing cases and we believe that the Street Outreach Office will start operating as soon as possible. Once it is up and running, it will greatly ease our burden on referrals. They will arrive either with the tests already prepared or with some pre-established diagnoses to help us with our work.* (I35)

## DISCUSSION

When understanding access and the dynamics of care for homeless persons, different situations and approaches are evident in the realities studied. However, in both scenarios, access occurs through spontaneous demand associated with professional attitudes that sometimes enhance access and sometimes limit it. Therefore, ensuring access is challenging, since there are several factors that can restrict or hinder care provision. Offering a service does not guarantee that persons will use it, since there are obstacles that prevent persons from accessing healthcare services, such as geographical barriers, travel problems, insufficient financial resources, and organizational and cultural barriers^(15)^. Thus, one of the challenges of caring for those who live on the streets is the lack of access to healthcare services, especially for those who use alcohol and other drugs and find themselves in a situation of extreme vulnerability^(16)^.

Due to the vulnerable conditions experienced by homeless persons, they have a disproportionate prevalence of chronic diseases when compared to other homeless persons. However, despite knowing their diagnosis, most of these persons do not receive follow-up or treatment for these diseases, which leads them to seek healthcare services in emergency situations to alleviate their symptoms. Thus, assessment and adaptation of existing services and initiatives to contemplate equity through technical, managerial and political actions within and between sectors will favor access, monitoring of chronic conditions and overcoming attitudes that stigmatize, exclude and promote the most diverse prejudices against persons living on the streets^(17)^. Furthermore, barriers to access to fundamental social rights and constitutional rights persist, despite the existence of sectoral and intersectoral policies aimed at protecting the rights of homeless persons. In this sense, the Social Assistance Network plays a fundamental role in the coordination and planning of the therapeutic journeys of these persons in the healthcare system, in order to guarantee their rights and strengthen the bond with the FHS teams^(18)^, which supports the findings of this study.

As evidenced in the scenarios studied, the *Centro POP* and Social Assistance Reference Center play a fundamental role in developing actions that favor the healthcare of these persons in the daily routine of FHS. In one of the municipalities, care was centralized in a FHS unit, which has the social assistance equipment in its area of coverage. However, it is essential to guarantee comprehensive, agile and timely access to the services and actions they need, since, after all, limiting care to a single health facility promotes inequality and goes against the PNPSR guidelines and recommendations. It is necessary to create mechanisms that facilitate the expansion of access to other health units, since the connection of homeless persons with the FHS team favors longitudinal monitoring^(1,5,17)^. Despite the difficulties, in the aforementioned service centralization unit, registered persons are linked to the “*Centro POP*” family, as defined by the participants of this study, in an intersectoral action between the FHS and the *Centro POP*, which strengthens the bond and provides access. In view of this, it is important to emphasize the importance of CHWs in registering users. This professional brings the community closer to the team. Whether the contact is at the user’s home, on the street or under the awning, it is in the territory that the needs and vulnerabilities of the persons who pass through and live there are identified^(15)^. In Natal, Rio Grande do Norte, Brazil, homeless persons “were classified as extra, floating and loose records” in the PHC registry^(19)^, a fact that requires rethinking and reorganizing current assistance practices, starting to consider them as subjects of rights.

The weakness in identifying and recognizing persons, regardless of whether they have a permanent residence in the area covered, means that they use emergency services as a gateway to the SUS, which indicates the need to overcome current practices. Due to the complexity of vulnerable conditions experienced, the reorganization of services will directly influence interventions and the determinants of the health-disease process, which is why it is necessary to readjust practices that require differentiated methods of care. After all, in practice, it is clear that “because someone does not belong to a defined territory, they easily end up belonging to no one”^(2)^.

In order to alleviate health inequities, care for homeless persons needs to be structured according to the needs imposed by the way of living on the streets and the establishment of links with PHC professionals. In this way, it will be possible to overcome the barriers that hinder these persons’ access to healthcare services, which is why professional training becomes an important challenge to be overcome when it comes to healthcare for persons in vulnerable conditions^(20)^. Professional unpreparedness is associated with discrimination and increased social vulnerability of these persons, in addition to low resolution in the face of the demands presented^(16)^.

In the reality of PHC, professionals focus on the needs of persons registered at the health unit and plan activities according to the profile of their community to ensure access and reduce inequities. This context makes it impossible to establish rules for standardizing care, even among similar services in different realities, which is why it is essential to develop initiatives that encourage more equitable care^(15)^. In the scenarios studied, actions are not planned to provide care to homeless persons. The practices carried out were closely related to specific actions in the places where these persons are served, such as the *Centro POP* and the Shelter House in the municipalities where this study was conducted. In this sense, it is necessary to overcome access barriers and maximize the effectiveness of actions that encourage the use of services, especially for more vulnerable populations^(18)^. One of the guidelines of PHC is to overcome health inequities, but its effectiveness depends on professionals’ behavior and proactivity for care quality and production. Therefore, discussions about healthcare planning are essential. It is important to take into account the needs and characteristics of persons living in the area covered. This requires planning and team engagement, with the aim of improving access, the work offered and the level of care provided as well as ensuring the satisfaction of these professionals with the work^(15)^. Hence, effective, comprehensive and quality care involves several actors: the professional, the team, the management and the user themselves^(21)^. Thus, to restructure PHC services, it is necessary to first analyze inclusion practices and what interferes so that exclusion occurs and, from this, build flows that organize actions that aim at universal access, with horizontal care in the RAS and co-responsibility by all those involved^(2)^.

A study conducted in four European countries revealed how stigmatizing and discriminatory attitudes by healthcare professionals negatively impact homeless persons’ health. It shows how the stigma experienced acts as a dissuasion for these persons to seek healthcare services, despite also presenting professional practices that have demonstrated positive results in providing care to these persons. Individualized approaches focused on persons’ needs have shown promise in this context. It is essential to exchange experiences and replicate best practices to ensure access to quality care, free from stigma and discrimination^(9)^.

Transformations that transcend traditional care practices are urgently needed to build strategies and possibilities that guarantee greater access to healthcare services for homeless persons^(6)^. Intersectoral partnerships allow for the development of new approaches and the reorganization of the health work process with equitable access practices^(20)^, in which knowledge complements each other and converges towards problem-solving in the daily lives of PHC professionals, which favors the incorporation of RAS in care planning and decision-making^(21)^.

Recognizing homeless persons in their subjectivities and specificities is essential to formulate the construction of care in their trajectories. Furthermore, it is urgent that the services provided offer the necessary care to promote subject autonomy in the face of an exclusionary and segregating society. In addition to the creation of specialized services, it will strengthen access to the promotion of intra and intersectoral network activities to strengthen relationships and mutual trust among the subjects involved^(16)^.

The PNPSR presents, among its objectives, the continuing training and qualification of professionals for developing actions and execution of transversal intersectoral policies aimed at persons living on the streets^(5)^. The introduction of SOOt changes the lack of accessibility by offering comprehensive care to the health needs of those living on the streets and increasing their access to care, accompanying them wherever they are^(20)^, in addition to developing integrated actions with the various services provided in the RAS^(16)^. It is worth noting that the implementation of SOOt is not the only way for these persons to access healthcare services and that care is the responsibility of PHC teams in municipalities that do not have this equipment, or even for those that have this service, but face organizational challenges and difficulties in articulating with intra and intersectoral services^(18)^.

Overcoming hygiene and welfare actions to project a perspective beyond prejudice, taking into account the physical and social vulnerabilities of persons living on the streets, will provide an equitable level of care. It is necessary to consider these persons as subjects who produce care, who constitute knowledge, and who will promote their potential and dignity^(22)^. Furthermore, interprofessional action enables sharing skills, favors the guarantee of comprehensive care and provides opportunities for improvements in healthcare practices as well as the possibility of facing problems arising from the care model and the workforce^(23)^.

Intersectoral actions between primary care services and the social sector to ensure adherence to treatment by homeless persons after hospital discharge are highlighted as relevant by a study conducted in a city in the state of New York^(10)^. This intersectoral collaboration, also addressed in this study, is configured as a link to ensure continuity of care, social reintegration and improvement of the quality of life of these persons. Hence, professional proactivity in the Social Assistance Network in caring for homeless persons is essential, as it addresses both immediate needs and systemic issues. It is necessary to provide equitable healthcare, provide conditions to address the structural causes of homelessness and recognize homelessness as an ethical challenge that involves rights, justice and equity^(24)^.

Proactive strategies significantly increase access to and quality of care. Moreover, they create a safe environment where homeless persons feel comfortable expressing their needs, which favors greater resolution in care. Thus, professional proactivity in offering clinical and integrated interventions not only improves the health outcomes of the person being treated, but also strengthens the link between these persons and the health and social assistance systems, with more comprehensive and effective support^(25)^.

From an interactionist perspective^(14)^, the meanings of this study translate into an action/performance/attitude for access to health for homeless persons, through the PHC door, in municipalities without SOOt. There is no longitudinality of care. Despite the defensive behavior of these persons, proactive attitudes of professionals minimize inequities and favor access, even in the face of centralization of care and fragility in recognizing these persons.

The challenge of providing healthcare to homeless persons is to offer assistance that goes beyond what is expected, planned and prescribed^(2)^, requiring professionals to have proactive, creative, innovative and unique attitudes^(6,26)^.

### Study limitation

As a limitation, one can consider the intentional sampling that focuses on municipalities in midwestern Minas Gerais without SOOt, but it was essential due to the evidence presented in different realities on the need to include homeless persons in the system through FHS/PHC as a universal and equitable right.

### Contributions to nursing, health and public policies

For the public agenda, this study points to the need for intersectoral action by presenting results of the integration of the Unified Social Assistance System and the SUS to guarantee access to PHC for homeless persons, with awareness among professionals regarding the specificities of these persons, i.e., singular and participatory care including the person being cared for. It highlights the need for strategies that enable progress in care for homeless persons in municipalities without SOOt, in order to combat social invisibility, expand access to health through PHC as a care coordinator, in search of guaranteeing the rights of persons living in vulnerable conditions.

## FINAL CONSIDERATIONS

This study showed that, although there is no SOOt in the municipalities studied, intersectoral actions associated with professional proactivity favor healthcare for homeless persons. Although care is provided in shelters, in spontaneous demand and aimed at acute conditions, some actions are specifically planned and developed with a view to preventing risks, reducing harm and promoting health. The interventions developed by the Social Assistance Network make it possible to reduce health inequities, given the compromise in guaranteeing fundamental rights, such as access to health, food and shelter.

The organization of work in PHC requires new approaches to care for persons in more vulnerable conditions, without centralizing care in a specific PHC unit, but rather on-site, with referrals, if necessary, and institutional inclusion. It is worth noting that planning intersectoral actions will help improve health conditions and strengthen the bond with PHC professionals. It is necessary to ensure access to health in the daily routine of PHC, in a way that expands it, considering the diversity in the territory and the most varied forms of occupation, with identification, registration and monitoring of persons living on the streets.

Organizing meetings and creating spaces for discussion about care for homeless persons in a network can make it possible to raise awareness among professionals about the importance of expanding access, reducing the barriers that make it difficult for these persons to access healthcare services, such as institutional prejudice and individual discrimination.

## Data Availability

Not applicable
