# Supplementary material for: Care for ill-housed persons: access and professional proactivity in primary care
Source: Rev Bras Enferm. 2025 Aug 25;78(Suppl 2):e20230385. doi: 10.1590/0034-7167-2023-0385 (PMC12380422; doi:10.1590/0034-7167-2023-0385)
Supplement: Translation

# Translation: Atenção às pessoas mal alojadas: acesso e proatividade profissional na atenção primária

**DOI:** 10.1590/0034-7167-2023-0385pt

**Authors:** Lucas Alves Gontijo, Bruna Moreira da Silva, Marcelo Pedra Martins Machado, Daniela Priscila Oliveira do Vale Tafner, Edilene Aparecida Araújo da Silveira, Valéria Conceição de Oliveira, Eliete Albano de Azevedo Guimarães, Selma Maria da Fonseca Viegas

**Affiliations:** I Universidade Federal de SÃ£o JoÃ£o del-Rei. DivinÃ³polis, Minas Gerais, Brazil.; II Fiocruz BrasÃ­lia. BrasÃ­lia, Distrito Federal, Brazil.; III Universidade Regional de Blumenau. Blumenau, Santa Catarina, Brazil.

**Keywords:** Pessoas Mal Alojadas, Atenção Primária à Saúde, Acesso aos Serviços de Saúde, Pessoal de Saúde, Equidade no Acesso aos Serviços de Saúde

## Abstract

**Objetivo:**

compreender o acesso à atenção primária de pessoas mal alojadas e a proatividade do profissional em municípios sem Consultório na Rua.

**Método:**

estudo de abordagem qualitativa, sob o referencial metodológico da Teoria Fundamentada nos Dados e referencial teórico do Interacionismo Simbólico, realizado com 30 profissionais da Estratégia Saúde da Família e seis informantes-chave da Rede Socioassistencial. Utilizaram-se a entrevista aberta e memorandos.

**Resultados:**

evidenciou-se a inexistência de planejamento da atenção à saúde de pessoas mal alojadas. O acesso se dá no acolhimento da demanda espontânea e, quase sempre, sob intervenção de profissionais da Rede Socioassistencial. Apontaramse a intersetorialidade e a proatividade do profissional enquanto eixos na superação das iniquidades e garantia do acesso.

**Considerações finais:**

ratifica-se a necessidade da ampliação do acesso, com identificação, cadastramento e acompanhamento das pessoas mal alojadas na atenção primária. É preciso estimular a proatividade do profissional, visto sua notoriedade no fortalecimento de ações equânimes.

## INTRODUÇÃO

Como uma porta de entrada preferencial no Sistema Único de Saúde (SUS), a Atenção Primária à Saúde (APS) ordena os fluxos das pessoas na Rede de Atenção à Saúde (RAS), a fim de garantir que a atenção seja ofertada próximo à casa de cada um. A APS tem como atributo a capacidade de atendimento às especificidades locais e características regionais, como a presença de populações itinerantes e dispersas na área de abrangência, garantindo, então, a efetividade da equidade em saúde^(1)^.

A saúde é um direito fundamental de cidadania, e isso implica que o Estado assegure a todos o acesso às ações e serviços de saúde por meio de políticas sociais e econômicas. Porém, muitos excluídos continuam lutando pelo direito de participar ativamente da sociedade, como é o caso das pessoas em situação de rua, que vivenciam estigmas e preconceitos, à margem de processos sociais. Além disso, experienciam diversos graus de violações e vulnerabilidades^(2,3)^, os quais constituem fatores que contribuem na legitimação da violência contra a identidade dessas pessoas^(4)^. Neste sentido, para assegurar o acesso amplo, simplificado e seguro às políticas públicas, foi instituída, no Brasil, em 2009, a Política Nacional para Pessoas em Situação de Rua (PNPSR)^(5)^, representando um avanço no atendimento dessas pessoas por meio de ações intersetoriais^(4)^, além de promover os princípios de equidade, humanização e universalidade, reconhecendo a singularidade e o respeito à dignidade humana e cidadania dessas pessoas^(6)^. Perante a nomenclatura adotada no Brasil, pessoas mal alojadas, nos Descritores em Ciências da Saúde e *Medical Subject Headings*, serão neste estudo denominadas de"pessoas em situação de rua".

De modo a colaborar na superação das iniquidades e ampliação do acesso à saúde das pessoas em situação de rua, o Ministério da Saúde instituiu, em 2011, a equipe de Consultório na Rua (eCR), que visa ampliar as respostas às demandas e necessidades dessas pessoas de forma itinerante e integrada com as equipes da Estratégia Saúde da Família (ESF) e, quando necessário, com outros pontos de atenção à saúde, de acordo com as demandas singulares^(7)^. No entanto, apesar dos avanços dos direitos adquiridos pelas pessoas em situação de rua, elas continuam enfrentando obstáculos para o acesso à saúde, principalmente em municípios sem eCR, fator que gera exclusão, restando o acesso às unidades de urgência e emergência^(2)^. Dessa forma, a colaboração com as redes intra e intersetorial se faz precisa, de modo a promover equidade e melhorias na condição de vida e no modo de viver nas ruas, com redução de danos e de riscos à saúde^(8)^.

Nas últimas décadas, as pessoas em situação de rua se tornaram um problema de saúde pública e política global. Estudo realizado na Áustria, Grécia, Espanha e Reino Unido evidenciou que a relação entre essas pessoas e a saúde é complexa, porque a falta de moradia é a causa de problemas de saúde e suas complicações que, por sua vez, aumentam o tempo na rua. Essas pessoas morrem mais cedo que a população geral, e têm menos acesso à APS, pois frequentam mais o serviço de urgência e emergência e nem sempre recebem tratamento e acompanhamento adequados para doenças crônicas. É urgente garantir acesso, cuidado e acolher as necessidades das pessoas que vivem nas ruas ao invés de enfrentarem um sistema de saúde complexo, segregador e excludente^(9)^.

Diante da complexa realidade das pessoas em situação de rua, marcada por necessidades sociais e clínicas exacerbadas, em Buffalo, Nova Iorque, foi criada uma equipe colaborativa, intersetorial, interagência e multidisciplinar, no intuito de apoiar a transição de cuidados a essas pessoas na alta hospitalar por meio de atenção médica e programa de descanso. O estudo aponta que o modelo organizacional tradicional dos serviços de saúde se mostra insuficiente, e é necessário um novo paradigma que transcenda as práticas curativistas e abrace um enfoque intersetorial e proativo, priorizando a colaboração entre diferentes setores e a atuação proativa dos profissionais envolvidos^(10)^.

## OBJETIVO

Compreender o acesso à atenção primária de pessoas mal alojadas e a proatividade do profissional em municípios sem Consultório na Rua.

## MÉTODOS

### Aspectos éticos

A pesquisa respeitou aspectos éticos das Resoluções no. 510/2016 e no. 580/2018 do Conselho Nacional de Saúde. A fim de garantir a privacidade dos participantes, foi assinado o Termo de Consentimento Livre e Esclarecido após pertinentes informações sobre a pesquisa e os preceitos éticos^(11,12)^. O projeto foi aprovado pelo Comitê de Ética em Pesquisa. Os participantes foram identificados com código alfanumérico (E_1_, E_2_, E_3_...).

### Tipo de estudo e referencial teórico-metodológico

Trata-se de estudo de abordagem qualitativa, resultante de uma dissertação de mestrado que teve como referencial metodológico a Teoria Fundamentada nos Dados (TFD)^(13)^ e referencial teórico o Interacionismo Simbólico (IS)^(14)^.

A TFD^(13)^ fornece uma combinação de resultados que permite explicar os fenômenos, além de discernir, compreender e formar um plano de ação ou teoria por meio do uso dos dados coletados^(12)^. O IS possibilita compreender como os indivíduos percebem os outros por meio de suas ações e interações e como ressignificam suas experiências coletivamente. Baseia-se em três premissas, que se concentram na compreensão do mundo social a partir das perspectivas das pessoas, a saber: a ação social é mediada por significados, e as pessoas agem com base no significado que as coisas têm para elas; os significados são emergentes da interação social e os significados são formados durante a interação; os significados são dinâmicos e interpretativos, ou seja, estão em constante mudança, e podem ser reinterpretados de acordo com o contexto e a situação^(14)^.

Para atender aos critérios no relato de pesquisa qualitativa, os pesquisadores adotaram o *COnsolidated criteria for REporting Qualitative research* (COREQ).

### Procedimentos metodológicos

A seleção das unidades de saúde da APS ocorreu por meio de sorteio aleatório, exceto as que se fazem referência para as pessoas que vivem nas ruas, sendo incluídas sem sorteio. Após abordagem inicial dos participantes, de forma presencial no local de trabalho com obtenção do Termo de Consentimento Livre e Esclarecido, procedeu-se à entrevista. Participaram deste estudo 30 profissionais da APS e seis informantes-chave, que são profissionais de instituições de acolhimento às pessoas em situação de rua com pelo menos um ano de exercício no cargo. Foram excluídos os profissionais afastados do trabalho ou de férias.

As entrevistas aconteceram presencialmente nas unidades de saúde e instituições de acolhimento em sala reservada, após o aceite voluntário do participante e posterior agendamento para realização da coleta de dados, sendo o número de participantes determinado pela saturação teórica dos dados, isto é, saturação dos conceitos/significados oriundos dos dados brutos^(13)^, totalizando 36 participantes da pesquisa.

### Cenário do estudo

O cenário do estudo constitui de dois municípios de médio porte pertencentes à Macrorregião Oeste do estado de Minas Gerais (MG), Brasil, sem eCR. O tamanho da população foi um fator decisivo no processo de seleção. Foram escolhidas as duas maiores cidades da região, conforme os números estimados pelo Instituto Brasileiro de Geografia e Estatística (IBGE) em 2022.

### Fonte de dados

Utilizou-se a entrevista aberta e intensiva, com roteiro semiestruturado e registro em memorandos.

### Coleta e organização dos dados

A coleta de dados ocorreu entre fevereiro e novembro de 2022. A entrevista foi realizada pelo pesquisador principal juntamente com aluna de iniciação científica, audiogravada e transcrita na íntegra com concomitante análise, conforme premissa da TFD. A entrevista foi validada pelo participante da pesquisa após leitura da transcrição, e todos os participantes deste estudo permitiram a utilização dos dados da sua entrevista em sua totalidade. A entrevista foi guiada por roteiro semiestruturado com nove questões que abordaram a atenção à saúde de pessoas em situação de rua, de forma a estimular a reflexão do participante sobre as experiências vivenciadas e relacionadas ao objeto em estudo. Com uma abordagem flexível, foi possível aprofundar os conceitos/ significados para o desenvolvimento da teoria. A duração média das entrevistas foi de 20 minutos, variando entre 13 e 42 minutos, de acordo com a liberdade de expressão ao abordar a temática e perante as experiências singulares vivenciadas.

### Análise dos dados

A análise dos dados se deu em quatro etapas, como codificações aberta, axial, seletiva e para o processo, permitindo maior flexibilidade na interpretação e no aprofundamento dos significados, a partir das realidades vivenciadas^(13)^.

Na codificação aberta, em um processo dinâmico e fluido, as falas dos entrevistados possibilitaram revelar, nomear e desenvolver conceitos que originaram os códigos in vivo e as propriedades. Na codificação axial, os dados obtidos na codificação aberta foram subcategorizados e incorporados em subtemáticas^(13)^, determinando cinco categorias: 1) Demanda espontânea, complexidade da atenção e proatividade profissional em municípios sem Consultório na Rua; 2) Exclusão, preconceito e invisibilidade de pessoas em situação de rua refutando o direito à saúde; 3) Equidade no enfrentamento das iniquidades: acolhimento de pessoas em situação de rua; 4) Dificuldades na implementação de estratégias de autocuidado para pessoas em situação de rua; 5) Na Atenção Primária, o encontro com as pessoas em situação de rua: como são e por que saíram de casa? Na codificação seletiva, foram realizadas a integração e a refinação da teoria, de modo a representar os resultados da pesquisa, assumindo então a categoria central "Atenção às pessoas em situação de rua em municípios sem Consultório na Rua: do acesso ao enfrentamento das iniquidades", também denominada de teoria. Durante todas essas etapas, ocorreu a codificação para o processo, que consistiu no ir e vir dos dados até o momento da construção da categoria central, e os dados foram contestados e analisados recorrentemente durante todo o processo de análise^(13)^.

## RESULTADOS

Foram entrevistados 36 profissionais, sendo sete enfermeiros, um auxiliar de enfermagem, três técnicos de enfermagem, três dentistas, um técnico de saúde bucal, dois auxiliares de saúde bucal, quatro médicos, nove Agentes Comunitários de Saúde (ACS), três psicólogos, um atendente de saúde bucal, um cuidador social e três assistentes social. Desses, 25 são do sexo feminino e 11, do sexo masculino, com a média de idade de 39 anos, com desvio padrão de 22 e 59 anos, e o tempo de atuação na área variou de 1 a 28 anos.

Este artigo aborda a categoria "Atenção às pessoas em situação de rua em municípios sem Consultório na Rua: acesso e proatividade profissional". Conforme visualizado na Figura 1, é possível identificar os códigos *in vivo*/propriedades, subcategorias e categoria que deram origem a este artigo.

**Figura 1:** Categoria "Atenção às pessoas em situação de rua em municípios sem Consultório na Rua: acesso e proatividade profissional", subcategorias e códigos *in vivo*, dois municípios da região Centro-Oeste de Minas Gerais, Brasil, 2022

*Nota: ^1^Centro de Referência Especializado para População em Situação de Rua;^2^Atenção Primária à Saúde.*

A atenção às pessoas em situação de rua apresenta singularidades, no cotidiano da APS, nos dois cenários deste estudo.

"Porta aberta", "é livre demanda", "chegou, a gente acolhe" são expressões dos participantes da pesquisa, ao enfatizar que as ações em saúde direcionadas às pessoas em situação de rua se voltam para a demanda espontânea em resposta a uma necessidade pontual:

*Na verdade, não tem um atendimento certo, porque é porta aberta* [...] *mas o atendimento de ir nas visitas, não tem! Porque eles não têm lugar onde morar. Aí é porta aberta quando eles sentem alguma necessidade de saúde, de atendimento, aí eles vêm na unidade. Não pode negar*. (E15)

*É a porta aberta. É a livre demanda deles. Quando eles precisam de alguma coisa da gente, a gente fornece a eles. Eles vêm, pedem e a gente fornece a ajuda que eles estão precisando*. (E16)

O acesso se dá no acolhimento à demanda espontânea, em horários pré-estabelecidos, por vezes em circunstâncias agudizadas:

*A demanda aqui, na verdade, ela é espontânea* [...] *porque eles só buscam quando eles estão numa situação muito precária, que é de dor, como no caso da dor de dente, ou quando tem alguma queda, ou se metem em alguma confusão que machuca, que tem que cuidar de uma ferida, de uma fratura, alguma coisa assim. Dificilmente, eles vêm de livre espontânea vontade.* (E26)

O acolhimento na APS é viabilizado via encaminhamentos realizados por profissionais da Rede Socioassistencial ou em comunicação prévia com o serviço para o qual o usuário é encaminhado.

*Morador de rua, geralmente, eles já veem com encaminhamento.* [...] *ele sempre é encaixado na agenda daquele dia. Então, o atendimento é tranquilo, ele não vai precisar esperar muito. Caso não tenha vaga naquele dia, a gente tenta marcar o mais próximo.* (E20)

Assim, nota-se que o atendimento à saúde se dá de forma centralizada em determinadas equipes de APS dos municípios, visto a existência de equipamentos socioassistenciais na área de abrangência que realizam a vinculação das pessoas em situação de rua aos serviços de APS:

*Aqui, o fluxo de atendimento, eles pertencem a uma área, então a microárea onde fica o núcleo POP tem uma ACS que tem o contato direto lá, tem o prontuário e, de forma geral, eles são encaminhados pela assistente social do núcleo POP ou eles ligam aqui na unidade e trazem a demanda.* (E8)

*Basicamente, aqui tem duas formas, demanda espontânea, que é a porta aberta, e tem a Casa de Acolhimento, que é aqui na nossa área de abrangência* [...] *quando ela* [assistente social] [...] *vê que precisa de algum atendimento da parte da saúde, ela entra em contato com a gente, e a gente normalmente agenda ou ela encaminha com encaminhamento simples.* (E31)

Segundo os participantes deste estudo, no cotidiano, eles enfrentam obstáculos significativos ao cuidar de pessoas em situação de rua, pela ausência de história de cuidado longitudinal e pelo fato de as intervenções ocorrerem, frequentemente, em situações tensas pelo comportamento defensivo dessas pessoas e efeitos de substâncias psicoativas. Esses obstáculos dificultam a articulação e a construção cotidiana dos planos de cuidado para essas pessoas no contexto da APS.

*Assim, a dificuldade maior que eu vejo é o primeiro contato com eles, da agressividade que eles vêm. Eles acham que a gente tem preconceito com eles.* (E3)

*É um público difícil. Às vezes, já chega aqui com algumas alterações; a maioria vai ter doença psiquiátrica; 99% vai ter algum tipo de dependência: álcool, drogas... então já é naturalmente difícil.* [...] *muita gente já chega alterado, aí, como você vai abordar esse público? É uma dificuldade, a pessoa já chega alterada, você não tem histórico, a pessoa chega com relato que roubaram a medicação ou sumiu, aí como você dá continuidade nisso?* [...] *há múltiplos fatores. É difícil fazer o acompanhamento longitudinal.* (E31)

São expressas reflexões sobre a melhoria do atendimento às pessoas em situação de rua, e são apontadas estratégias para ampliar o acesso e melhorar a atenção à saúde dessas pessoas, por meio de atitudes profissionais proativas que minimizem as iniquidades em saúde.

*Fazer um mutirão, para ir até essa pracinha, com médico e enfermeira.* [...] *se formasse uma equipe que fosse até o encontro deles, um dia, uma vez no mês, que fosse até onde eles ficam localizados, ajudaria bastante.* (E15)

*Da saúde ir até onde o usuário está, porque, assim, muitas vezes, a gente não consegue sensibilizá-lo para ir à ESF. Talvez esse seria o caminho para conseguir equidade.* (E18)

*No quesito saúde, a gente poderia melhorar em projetos. Sei lá, colocar uma ambulância à noite, onde eles aglomeram mais... medir uma pressão, uma glicose. Ver como estão...* (E34)

Apesar da complexidade do atendimento às pessoas em situação de rua, fica claro nas falas dos participantes que a proatividade do profissional proporciona vínculos intra e intersetoriais na superação de barreiras e garantia do acesso ao cuidado. Este fato fica evidente na rotina dos serviços, quando inserem essas pessoas no cotidiano da APS, porém, no que tange ao cadastramento das pessoas na APS, os discursos divergem entre os participantes e os municípios.

*Ele tem o prontuário na unidade,* [...] *mas ele não está cadastrado. Ele não está vinculado a uma família.* (E7)

*É como se fosse uma família POP, sabe?* [...] *eles são cadastrados lá como uma família.* (E13)

*Pelo nosso método de trabalho, a gente cadastra as famílias de acordo com o endereço, e essas pessoas não têm como fazer isso.* (E16)

Objetiva-se assegurar a cada pessoa uma vida digna, incluindo saúde, alimentação e teto. Nesse sentido, o Centro de Referência Especializado para População em Situação de Rua (Centro POP) foi mencionado entre os participantes da pesquisa várias vezes:

*Então, hoje você pode ficar fixo na casa* [Centro POP] *ou você pode ir lá tomar banho, comer e voltar pra rua.* (E1)

*Tem o POP, que acolhe esses moradores de rua, os acompanha nas consultas especializadas, digo, assim, consulta na policlínica.* (E7)

Cada município sem eCR tem uma rotina e prática para o atendimento às pessoas em situação de rua, o que infere a garantia de acesso à saúde por meio de programas de assistência social que estabeleçam relações intersetoriais com unidades de saúde mais próximas da instituição de acolhimento, porém confere a centralização do atendimento em uma única unidade, ou em unidade mais central do município, onde circulam as pessoas em situação de rua como um "ponto de referência" na assistência à saúde dessas pessoas (memorando).

*Eles centralizaram o atendimento da população de rua na unidade que está na abrangência do núcleo POP. Então, hoje, o atendimento da população de rua que tem cadastro no núcleo POP é feito pela nossa unidade de saúde.* (E8)

*Aqui, a gente tem uma Casa de Acolhimento* [Centro de Referência de Assistência Social] *no centro, então, quando esse usuário chega lá e ele precisa do atendimento, então a própria casa o encaminha para cá.* (E28)

Em relação ao planejamento da assistência à saúde às pessoas em situação de rua, foi comum em ambas as realidades a execução de ações pontuais, de acordo com a necessidade demandada por elas e pelos serviços socioassistenciais, operando atendimentos às queixas pontuais, abstendo o desenvolvimento de ações planejadas, de modo a promover a saúde dessas pessoas, que possam impactar sobre os fatores que interferem na qualidade de vida e saúde.

*Não tem planejamento. Não existe. Depende da necessidade deles quando eles procuram a unidade. Mas a unidade fazer alguma coisa para promover? Não faz.* (E15)

*Não tem como planejar ação com essa população. É uma população muito flutuante, muito irregular.* [...] *nós já fizemos, de ir ali na Casa de Acolhimento,* [...] *fazer uma palestra com eles sobre autocuidado, sobre higienização, higiene bucal. É o que a gente consegue fazer até o momento,* [...] *não tem programações específicas para ir frequentemente, não. A gente foi porque eles nos convidaram.* (E33)

No entanto, mesmo sem planejamento da assistência à saúde de pessoas em situação de rua, identifica-se que as ações intersetoriais são extremamente importantes na construção do cuidado ofertado, visto que as equipes da APS se apresentam engajadas e solidárias ao atendimento dessas pessoas.

*Especificamente aqui no Centro POP, que é um serviço da assistência social, a questão de saúde, a gente tem mais uma ideia do apoio, a gente faz os encaminhamentos para a APS, para o hospital…* [...] *a gente constrói junto com o usuário um projeto, um plano de acompanhamento. Se a gente identifica alguma questão de saúde, é aquela questão do apoio. A gente apoia a pessoa, muitas vezes, acompanha as pessoas aos exames que ela tem que fazer, ao atendimento que ela tem que fazer na APS, os encaminhamentos para os demais serviços, tipo o CAPS.* (E17)

*Aqui, a gente tem muita facilidade em questão de saúde. A gente tem uma parceria muito boa com o pessoal do posto de saúde ali, tudo que a gente precisa, eles são atendidos, são encaminhados para lá.* [...] *tem, também, acompanhamento CAPS-AD. Alguns aqui frequentam, não são todos, e, quando frequentam, têm um amparo lá do CAPS-AD, mas é bem tranquilo. Quando é caso de urgência e emergência, a gente chama o SAMU, eles vêm.* (E36)

Um dos municípios está em processo de credenciamento de eCR. Diante dessa realidade, os profissionais se mostraram apreensivos e esperançosos com a implantação da equipe, devido à possibilidade de expansão do acesso e ao potencial de monitoramento longitudinal:

*Já está anunciado na imprensa o Consultório na Rua* [...] *seria uma unidade absolutamente importante para acompanhamento deles.* [...] *eu temo que o Consultório na Rua venha e vire um grande ambulatório, sem a vinculação psicossocial, sem a vinculação no território, sem a vinculação de geração de renda, sem outras possibilidades que essa população teria no território.* (E22)

*Tem feito levantamentos e discussões de casos, ea gente acredita que o mais breve possível o Consultório na Rua vai começar a funcionar. Funcionando já vai nos aliviar muito na questão de encaminhamentos. Eles já chegarão ou com os exames já prontos ou com alguns diagnósticos já pré-estabelecidos para nos ajudar no trabalho.* (E35)

## DISCUSSÃO

Ao compreender o acesso e a dinâmica do acolhimento das pessoas em situação de rua, evidenciam-se diferentes situações e abordagens nas realidades pesquisadas. No entanto, em ambos os cenários, o acesso ocorre por demanda espontânea associada a atitudes profissionais que ora facilitam o acesso ora o limitam. Destarte, garantir o acesso é desafiador, já que existem diversos fatores que possam restringir ou prejudicar a prestação do cuidado. Ofertar um serviço não garante que as pessoas o utilizem, pois existem obstáculos que impedem o acesso das pessoas aos serviços de saúde, como barreiras geográficas, problemas de deslocamento, insuficiência de recursos financeiros, barreiras organizacionais e culturais^(15)^. Assim, um dos desafios do cuidado de quem vive na rua é a falta de acesso aos serviços de saúde, principalmente para quem faz uso de álcool e outras drogas e se encontra em situação de vulnerabilidade extrema^(16)^.

Devido às condições vulneráveis vivenciadas pelas pessoas em situação de rua, elas têm uma prevalência desproporcional de doenças crônicas, quando comparadas às outras pessoas com teto. No entanto, apesar de conhecerem o diagnóstico, a maioria dessas pessoas não é acompanhada ou trata essas doenças, o que as leva a procurar os serviços de saúde em situações de urgência e emergência para aliviar os sintomas. Nesse sentido, a avaliação e a adequação dos serviços e iniciativas existentes para contemplar a equidade por meio de ações técnicas, gerenciais e políticas intra e intersetoriais favorecerão o acesso, o acompanhamento das condições crônicas e a superação de atitudes que estigmatizam, excluem e promovem os mais diversos preconceitos contra as pessoas que vivem nas ruas^(17)^. Além disso, as barreiras de acesso aos direitos sociais fundamentais e aos direitos constitucionais persistem, apesar da existência de políticas setoriais e intersetoriais voltadas para a proteção dos direitos da pessoa em situação de rua. Neste sentido, a Rede Socioassistencial desempenha um papel fundamental na articulação e no planejamento das jornadas terapêuticas dessas pessoas no sistema de saúde, de modo a garantir os seus direitos e o fortalecimento do vínculo com os profissionais das equipes da ESF^(18)^, o que corrobora os achados deste estudo.

Como evidenciado nos cenários estudados, o Centro POP e o Centro de Referência de Assistência Social têm papel fundamental no desenvolvimento de ações que favorecem o cuidado à saúde dessas pessoas no cotidiano da ESF. Em um dos municípios, o atendimento foi centralizado em unidade da ESF, que tem em sua área de abrangência o equipamento de assistência social. Porém, é fundamental garantir o acesso de forma integral, ágil e oportuno aos serviços e ações de que necessitam, pois, afinal, limitar o atendimento a um único estabelecimento de saúde promove a desigualdade e contraria as próprias diretrizes e recomendações da PNPSR. É preciso criar mecanismos que facilitem a expansão do acesso a outras unidades de saúde, já que a vinculação da pessoa em situação de rua com a equipe da ESF favorece o acompanhamento longitudinal^(1,5,17)^. Apesar das dificuldades, na referida unidade de centralização do atendimento, as pessoas cadastradas são vinculadas na família "Centro POP", conforme definido pelos participantes deste estudo, em uma ação intersetorial entre ESF e Centro POP, o que fortalece o vínculo e proporciona acesso. Diante disso, é importante ressaltar a importância do ACS quanto ao cadastramento dos usuários. Esse profissional aproxima a comunidade à equipe. Seja o contato na casa do usuário, na rua ou embaixo da marquise, é no território que são identificadas as necessidades e vulnerabilidades das pessoas que ali passam e residem^(15)^. Em Natal, Rio Grande do Norte, Brasil, as pessoas em situação rua"eram classificadas como ficha extra, flutuante e avulsa" no cadastramento da APS^(19)^, fato que exige repensar e reorganizar as práticas assistenciais vigentes, passando a considerá-los enquanto sujeitos de direitos.

A fragilidade na identificação e no reconhecimento de pessoas, independentemente da existência de moradia fixa na área de abrangência, faz com que usufruem de serviço de urgência e emergência como porta de entrada no SUS, o que sinaliza a necessidade de superação das práticas atuais. Devido à complexidade de condições vulneráveis vivenciadas, a reorganização dos serviços influenciará diretamente as intervenções e os determinantes do processo saúde-doença, por isso é preciso readequar as práticas, que requerem métodos diferenciados no atendimento. Afinal, na prática, percebe-se que "por não pertencer a um território definido, facilmente acaba por pertencer a ninguém"^(2)^.

A fim de amenizar as iniquidades em saúde, a atenção às pessoas em situação de rua precisa ser estruturada de acordo com as necessidades impostas pelo modo de viver na rua e o estabelecimento de vínculos com os profissionais da APS. Destarte, será possível transpor as barreiras que dificultam o acesso dessas pessoas aos serviços de saúde, razão pela qual a capacitação profissional se torna um importante desafio a ser superado quando se trata de atenção à saúde de pessoas em condições vulneráveis^(20)^. O despreparo profissional está associado à discriminação e ao aumento da vulnerabilidade social dessas pessoas, além de baixa resolutividade diante das demandas apresentadas^(16)^.

Na realidade da APS, os profissionais têm como foco as necessidades das pessoas cadastradas na unidade de saúde e planejam as atividades de acordo com o perfil de sua comunidade para garantir o acesso e a diminuição das iniquidades. Esse contexto impossibilita o estabelecimento de regras de uniformização de atendimentos, mesmo entre serviços semelhantes em realidades díspares, razão que torna imprescindível desenvolver iniciativas que estimulem o cuidado mais equitativo^(15)^. Nos cenários pesquisados, as ações não são planejadas para a atenção às pessoas em situação de rua. As práticas realizadas estavam intimamente relacionadas às ações pontuais nos locais onde essas pessoas são atendidas, como o Centro POP e a Casa de Abrigo dos municípios cenários deste estudo. Nesse sentido, precisa-se superar as barreiras de acesso e maximizar a efetividade de ações que estimulem a utilização dos serviços, principalmente para populações mais vulneráveis^(18)^.

A APS tem como uma de suas diretrizes a superação das iniquidades em saúde, mas sua efetividade interdepende do comportamento e proatividade dos profissionais para a qualidade da atenção e produção do cuidado. Por isso, discussões sobre planejamento da atenção à saúde são essenciais. É importante levar em consideração as necessidades e características das pessoas que vivem na área de abrangência. Isso requer planejamento e engajamento da equipe, a fim de melhorar o acesso, o trabalho oferecido e o nível de atenção prestado, bem como garantir a satisfação desses profissionais com o trabalho^(15)^. Nesse sentido, uma assistência resolutiva, integral e de qualidade envolve vários atores: o profissional, a equipe, a gestão e o próprio usuário^(21)^. Assim, para reestruturação dos serviços de APS, é preciso, primeiramente, analisar as práticas de inclusão e o que interfere, para que a exclusão aconteça e, a partir disso, construir fluxos que organizem ações que visem à universalidade do acesso, com cuidado horizontal na RAS e corresponsabilizado por todos os envolvidos^(2)^.

Estudo realizado em quatro países da Europa revelou como as atitudes estigmatizantes e discriminatórias por parte de profissionais de saúde impactam negativamente a saúde de pessoas em situação de rua. Demonstra como o estigma vivenciado atua como um dissuasivo para essas pessoas buscarem os serviços de saúde, apesar de apresentar também práticas profissionais que demonstraram resultados positivos no atendimento a essas pessoas. Abordagens individualizadas voltadas para as necessidades das pessoas mostraram-se promissoras nesse contexto. Torna-se fundamental a troca de experiências e a replicação das melhores práticas para garantia do acesso a um atendimento de qualidade, livre de estigmas e discriminação^(9)^.

Transformações que transpassem as práticas assistenciais tradicionais se tornam urgentes, de modo a construir estratégias e possibilidades que garantam maior acesso aos serviços de saúde pelas pessoas em situação de rua^(6)^. As articulações intersetoriais permitem o desenvolvimento de novas abordagens e reorganização do processo de trabalho em saúde com práticas de acesso equitativo^(20)^, em que os saberes se completam e convergem para a resolutividade no cotidiano dos profissionais da APS, o que favorece a incorporação da RAS no planejamento do cuidado e na tomada de decisão^(21)^.

Reconhecer as pessoas em situação de rua em suas subjetividades e especificidades é fundamental para formular a construção do cuidado em suas trajetórias. Além disso, é urgente que os atendimentos prestados ofereçam o cuidado necessário para promover a autonomia do sujeito diante de uma sociedade excludente e segregadora. Além de criar serviços especializados, fortalecerá o acesso à promoção de atividades em rede intra e intersetorial, para fortalecer as relações e a confiança mútua entre os sujeitos envolvidos^(16)^.

A PNPSR apresenta, entre os seus objetivos, a formação e a capacitação permanente dos profissionais para o desenvolvimento de ações e execução de políticas intersetoriais transversais direcionadas às pessoas em situação de rua^(5)^. A introdução de eCR vem alterando o quadro de falta de acessibilidade, ao oferecer atendimento integral às necessidades de saúde daqueles que vivem nas ruas e aumentar seu acesso aos cuidados, acompanhando-os onde quer que estejam^(20)^, além de desenvolver ações integradas com os diversos serviços dispostos na RAS^(16)^. Vale ressaltar que a implantação da eCR não é a única forma dessas pessoas acessarem os serviços de saúde e que o cuidado é de responsabilidade das equipes da APS nos municípios que não possuem esse equipamento, ou até mesmo para aqueles que têm este serviço, mas enfrentam desafios organizacionais e dificuldades de articulação com os serviços intra e intersetoriais^(18)^.

A superação de ações higienistas e assistencialistas para projetar o olhar além do preconceito, em consideração às vulnerabilidades físicas e sociais de pessoas vivendo nas ruas, proporcionará um nível equitativo de cuidado. Torna-se preciso considerar essas pessoas como sujeitos produtores de cuidado, constituintes de saberes, promovendo suas potencialidades e dignidade^(22)^. Além disso, a atuação interprofissional possibilita o compartilhamento de habilidades, favorece a garantia da integralidade no atendimento e viabiliza oportunidades de melhorias nas práticas da assistência à saúde, como também na possibilidade de enfrentar os problemas decorrentes do modelo de atenção e da força de trabalho^(23)^.

As ações intersetoriais entre os serviços de cuidados primários e o setor social, para adesão ao tratamento pelas pessoas em situação de rua no pós-alta hospitalar, são apontadas como relevantes por estudo realizado em cidade do estado de Nova Iorque^(10)^. Essa colaboração intersetorial, abordada também neste estudo, se configura como um elo para garantir a continuidade do cuidado, a reintegração social e a melhoria da qualidade de vida dessas pessoas. Neste sentido, a proatividade dos profissionais da Rede Socioassistencial e de saúde na atenção às pessoas em situação de rua é essencial, pois aborda tanto necessidades imediatas quanto questões sistêmicas. É preciso fornecer cuidados de saúde equitativos, propiciar condições de enfrentamento das causas estruturais da falta de moradia e reconhecer a situação de rua como um desafio ético que envolve direitos, justiça e equidade^(24)^.

As estratégias proativas aumentam significativamente o acesso e a qualidade do cuidado recebido. Além disso, cria um ambiente de segurança, onde as pessoas em situação de rua se sintam à vontade para expressar suas necessidades, o que favorece maior resolutividade no atendimento. Assim, a proatividade dos profissionais ao oferecer intervenções clínicas e integradas, não apenas melhora os resultados de saúde da pessoa atendida, mas também fortalece o vínculo entre essas pessoas e os sistemas de saúde e a assistência social, com um suporte mais abrangente e eficaz^(25)^. Na perspectiva interacionista^(14)^, os significados deste estudo traduzem uma ação/atuação/atitude para o acesso à saúde de pessoas em situação de rua, pela porta da APS, em municípios sem eCR. Não há longitudinalidade do cuidado. Apesar do comportamento defensivo dessas pessoas, atitudes proativas de profissionais minimizam as iniquidades e favorecem o acesso, mesmo diante da centralização da atenção e da fragilidade no reconhecimento dessas pessoas.

O desafio da atenção à saúde de pessoas em situação de rua está em oferecer uma assistência que vá além do que se espera, do que se planeja e do que se prescreve^(2)^, requerendo dos profissionais atitudes proativas, criativas, inovadoras e singulares^(6,26)^.

### Limitação do estudo

Como limitação, pode-se considerar a amostragem intencional que se concentra em municípios da região Centro-Oeste de Minas Gerais sem eCR, mas se fez essencial pelas evidências apresentadas em realidades distintas sobre a necessidade de inclusão das pessoas em situação de rua no sistema pela porta da ESF/APS como direito universal e equânime.

### Contribuições para as áreas da enfermagem, saúde e políticas públicas

Para a agenda pública, este estudo aponta para a necessidade da intersetorialidade, ao apresentar resultados de integração do Sistema Único da Assistência Social e do SUS para garantia do acesso à APS de pessoas em situação de rua, com sensibilização dos profissionais quanto às especificidades dessas pessoas, ou seja, a assistência singular e participativa incluindo a pessoa cuidada. Traz a necessidade de estratégias que possibilitem avançar na atenção às pessoas em situação de rua, em municípios sem eCR, de modo a combater a invisibilidade social, ampliar o acesso à saúde pela APS como coordenadora do cuidado, em busca da garantia de direitos de pessoas que vivem em condições de vulneração.

## CONSIDERAÇÕES FINAIS

Este estudo mostrou que, ainda que inexista a eCR nos municípios estudados, ações intersetoriais associadas à proatividade do profissional favorecem a atenção à saúde de pessoas em situação de rua. Embora os atendimentos ocorram no acolhimento, em demanda espontânea e voltados às condições agudizadas, algumas ações são pontualmente planejadas e desenvolvidas com vistas à prevenção de riscos, redução de danos e promoção da saúde. As intervenções desenvolvidas pela Rede Socioassistencial possibilitam a redução das iniquidades em saúde, visto o comprometimento na garantia dos direitos fundamentais, como o acesso à saúde, à alimentação e ao teto.

A organização do trabalho na APS necessita de novas abordagens no atendimento de pessoas em condições mais vulneráveis, sem centralização da atenção em unidade de APS específica, mas sim *in loco*, com encaminhamentos, se forem necessários, e inclusão institucional. Vale ressaltar que o planejamento das ações intersetoriais auxiliará em melhorias das condições de saúde e no fortalecimento do vínculo junto aos profissionais da APS. É preciso assegurar o acesso à saúde no cotidiano da APS, de modo que o amplie, considerando as diversidades no território e as mais variadas formas de ocupação, com identificação, cadastramento e acompanhamento de pessoas em situação de rua.

A articulação de encontros e a formação de espaços de discussão sobre a atenção às pessoas em situação de rua em rede podem viabilizar a conscientização dos profissionais sobre a importância da ampliação do acesso, diminuindo as barreiras que dificultam a chegada dessas pessoas no serviço de saúde, como o preconceito institucional e a discriminação singular.

## Data Availability

Não se aplica
